# Genomic monitoring to understand the emergence and spread of Usutu virus in the Netherlands, 2016–2018

**DOI:** 10.1038/s41598-020-59692-y

**Published:** 2020-02-18

**Authors:** B. Bas Oude Munnink, E. Münger, D. F. Nieuwenhuijse, R. Kohl, A. van der Linden, C. M. E. Schapendonk, H. van der Jeugd, M. Kik, J. M. Rijks, C. B. E. M. Reusken, M. Koopmans

**Affiliations:** 1 ErasmusMC, Department of Viroscience, WHO collaborating centre for arbovirus and viral hemorrhagic fever Reference and Research, Rotterdam, the Netherlands; 2 Vogeltrekstation - Dutch Centre for Avian Migration and Demography, NIOO-KNAW, Wageningen, the Netherlands; 3 Dutch Wildlife Health Centre, University of Utrecht, Utrecht, the Netherlands; 4 Department of Pathobiology, Pathology, Faculty of Veterinary Medicine, Utrecht University, Utrecht, the Netherlands; 5 Present Address: Centre for Infectious Disease Control, National Institute for Public Health and the Environment, Bilthoven, the Netherlands

**Keywords:** Evolutionary genetics, Next-generation sequencing

## Abstract

Usutu virus (USUV) is a mosquito-borne flavivirus circulating in Western Europe that causes die-offs of mainly common blackbirds (*Turdus merula*). In the Netherlands, USUV was first detected in 2016, when it was identified as the likely cause of an outbreak in birds. In this study, dead blackbirds were collected, screened for the presence of USUV and submitted to Nanopore-based sequencing. Genomic sequences of 112 USUV were obtained and phylogenetic analysis showed that most viruses identified belonged to the USUV Africa 3 lineage, and molecular clock analysis evaluated their most recent common ancestor to 10 to 4 years before first detection of USUV in the Netherlands. USUV Europe 3 lineage, commonly found in Germany, was less frequently detected. This analyses further suggest some extent of circulation of USUV between the Netherlands, Germany and Belgium, as well as likely overwintering of USUV in the Netherlands.

## Introduction

Usutu virus (USUV) is a mosquito-borne arbovirus of the genus *Flavivirus*. The virus has a positive-stranded RNA genome with a genome length of around 11,000 nucleotides which encodes a single polyprotein. The polyprotein is processed by viral and host proteases into structural and non-structural proteins^1^. The life cycle of USUV involves mosquitoes (mainly *Culex* sp.^2,3^) as vectors and wild birds as the main amplifying hosts. Humans and other mammals can be infected by mosquito bites and are generally considered dead-end hosts. Recent studies revealed that USUV can also be detected in small mammals such as bats^4^, rodents and shrews^5^.

USUV was first identified from a *Culex neavei* mosquito in South Africa in 1959^6^. Over subsequent decades, it was sporadically reported in several mosquito and bird species, and twice in a human patient with fever and rash in African countries^7^. USUV was for first identified in Europe in 2001, when it was determined to be the causative agent of a mass mortality event in several bird species in Austria^8^. This prompted retrospective analysis of archived tissue samples from dead wild birds in Italy in 1996, which revealed an earlier presence of the virus in Europe^9^. Our understanding of the USUV geographical range has since expanded to include the majority of European countries^10^, where outbreaks are marked by mass die-offs of wild birds, with the heaviest toll on common blackbird (*Turdus merula*) populations. In Germany, it has been demonstrated that five years after the first detection of USUV in the southwest of the country, circulation of the virus was associated with a 15.7% decline in the common blackbird population in USUV-suitable areas^11^.

The recent emergence of USUV epizootics among wild bird populations in Europe has been accompanied by reports of USUV infections in humans. Pathogenicity in humans appears to range from asymptomatic or mild symptoms, as shown in seroprevalence studies among healthy blood donors in Italy^12^, Germany^13^, Austria^14^ and the Netherlands^15^ to neuroinvasive infections associated with encephalitis or meningo-encephalitis, mainly in patients with underlying chronic disease or in immunocompromised patients, as described in Italy^16–19^, France^20^ and Croatia^21^. The number of human USUV cases described is very limited, but as it is likely that some clinical cases of acute USUV infection in humans remain remain undiagnosed, it is difficult to evaluate the real public health impact of USUV emergence in Europe at this stage. The circulation of USUV in birds can be linked to an increased human exposure to zoonotic risk. Indeed, the detection of viremic blood donations in the Netherlands co-incided with months of active USUV circulation in birds^22^, human cases described in Italy were from an area with demonstrated concomitant circulation of USUV in mosquitoes and birds^16–19^, and in Austria an increase in human cases was observed alongside the increased USUV activity in birds^23^.

Various USUV lineages are co-circulating in Europe^4,24^. In Germany, circulation of five different USUV lineages (Europe 2, Europe 3, Europe 5, Africa 2, and Africa 3) has been described^25,26^ and in France two lineages (Africa 2 and Africa 3) were detected in mosquitoes from the same region^27,28^. The co-circulation of different USUV lineages is thought to be due to independent introduction events to Europe by long-distance migratory birds from Africa, where different USUV lineages are presumed to be circulating, followed by local amplification and evolution leading to a geographical signal in phylogenetic analyses^29^. The assignment and nomenclature of USUV lineages are not standardized, and it is unclear if the lineages differ in their potential to be transmitted by mosquitoes and to infect or cause disease in different wild bird species and/or humans^29^.

In the Netherlands, USUV was first detected in 2016 when it was identified as the cause of an outbreak among blackbirds and captive great grey owls (*Strix nebulosa*)^28,30^. The virus circulated also in 2017 and 2018, and each late summer to autumn was associated with an increased die-off in blackbirds. Through a national wildlife disease surveillance programme, dead birds reported by citizens were collected, dissected and tissues were submitted for USUV diagnostics. To date, only a limited number of USUV genomes are available in the public domain, and it is unknown if USUV was already present in the Netherlands before 2016. In addition, it is unknown if there was a single introduction event or several independent introduction events, and – if so – what the geographic origin of the viruses is. Recent advances in third generation sequencing technologies have opened up new opportunities for infectious disease research. Pathogen genomics can be used to resolve crucial questions regarding origin, modes of transmission and ecology of emerging viral disease^31–35^. Therefore, we have sequenced 112 USUV genomes from blackbirds brain tissues on the Nanopore sequencing platform to analyse the emergence and spread of USUV throughout the Netherlands in 2016, 2017 and 2018.

## Results

### USUV genomic sequencing

Between September 2016 and September 2018, 165 dead blackbirds were screened for the presence of USUV by RT-PCR. This screening resulted in 118 USUV positive birds and genomic sequences were generated with an USUV specific multiplex PCR using the Nanopore sequencing technology. Successful sequencing was defined as obtaining at least 80% of the USUV genome with a read coverage threshold of 100x per amplicon^36^. Near complete or complete viral genomes were recovered from 112 of 118 samples (Table 1). The median Ct value of the USUV positive brain tissues was 20, and the threshold for successful sequencing directly from brain tissue samples was shown to be around Ct value 32. Above this Ct value, only some amplicons were sequenced with a coverage >100x. An overview of the number of reads generated, the percentage of USUV reads and the proportion of successfully sequenced amplicons is displayed in Supplementary Table 1.

**Table 1:** Overview of the different USUV blackbird samples sequenced during this study.

Sample ID	Accession number	Ct Value	Collection date	Location	USUV lineage
AS201600034	MN122145	19.45	01-09-16	Gennep	Africa 3.2
AS201600036	MN122146	15.38	06-09-16	Andelst	Africa 3.3
AS201600042	MN122147	14.21	07-09-16	Venlo	Africa 3.3
AS201600045	MN122148	14.97	07-09-16	Lierop	Africa 3.2
AS201600048	MN122149	19.74	07-09-16	Lierop	Africa 3.3
AS201600051	MN122150	21.44	07-09-16	Ottersum	Africa 3.3
AS201600057	MN122151	15.6	08-09-16	Sint Agatha	Africa 3.2
AS201600060	MN122152	19.75	12-09-16	Zevenaar	Europe 3
AS201600062	MN122153	16.99	13-09-16	Andelst	Africa 3.3
AS201600089	MN122154	21.08	20-09-16	Vlijmen	Europe 3
AS201600095	MN122155	19.78	21-09-16	Klarenbeek	Africa 3.3
AS201600098	MN122156	20.73	21-09-16	Uden	Africa 3.2
AS201600101	MN122157	20.34	22-09-16	Beek en Donk	Africa 3.3
AS201600104	MN122158	18.92	22-09-16	Heino	Europe 3
AS201600107	MN122159	21.85	22-09-16	Wernhout	Africa 3.3
AS201600110	MN122160	24.21	22-09-16	Reek	Africa 3.3
AS201600115	MN122161	24.06	22-09-16	Houten	Africa 3.3
AS201600118	MN122162	21.02	22-09-16	De Bilt	Africa 3.2
AS201600121	MN122163	20.39	22-09-16	Haarzuilens	Africa 3.2
AS201600124	MN122164	23.8	23-09-16	Westervoort	Africa 3.2
AS201600127	MN122165	17.47	23-09-16	Deventer	Africa 3.3
AS201600133	MN122166	22.59	23-09-16	Overdinkel	Europe 3
AS201600136	MN122167	16.99	23-09-16	Arnhem	Africa 3.3
AS201600148	MN122168	24.79	23-09-16	Nieuwegein	Africa 3.3
AS201600173	MN122169	20.33	07-10-16	Doetichem	Africa 3.2
AS201600197	MN122170	20.13	11-10-16	Reimerswaal	Europe 3
AS201600203	MN122171	32.01	05-10-16	Lelystad	Europe 3
AS201600221	MN122172	19.95	22-09-16	Haarzuilens	Europe 3
AS201600227	MN122173	26.23	30-09-16	Hilversum	Africa 3.3
AS201600241	MN122174	20.16	27-09-16	Midden-Drenthe	Europe 3
AS201600244	MN122175	18.89	27-09-16	Zutphen	Africa 3.2
AS201600247	MN122176	14.07	30-09-16	Terneuzen	Europe 3
AS201600253	MN122177	21.51	27-09-16	Landgraaf	Europe 3
AS201600265	MN122178	16.48	30-09-16	Enschede	Africa 3.3
AS201600268	MN122179	16.72	30-09-16	Nederlek	Europe 3
AS201600274	MN122180	21.11	27-09-16	streek De Liemers	Africa 3.3
AS201600277	MN122181	17.43	27-09-16	De Ronde Venen	Europe 3
AS201600280	MN122182	21.87	26-09-16	Ingen	Africa 3.2
AS201600281	MN122183	14.07	25-09-16	Langbroek	Africa 3.3
AS201600283	MN122184	19.94	27-09-16	Oss	Africa 3.3
AS201600284	MN122185	20.09	03-10-16	Oss	Africa 3.2
AS201600286	MN122186	28.48	28-09-16	Oss	Africa 3.2
AS201600287	MN122187	14.46	27-09-16	Schaik	Africa 3.2
AS201700024	MN122188	19.88	11-04-17	Westvoort	Europe 3
AS201700077	MN122189	16.8	03-07-17	Best	Europe 3
AS201700080	MN122190	19.6	05-07-17	Bilthoven	Europe 3
AS201700084	MN122191	16.6	05-07-17	Bunnik	Africa 3.3
AS201700086	MN122192	18.3	11-07-17	Best	Europe 3
AS201700087	MN122193	17	11-07-17	Zutphen	Africa 3.3
AS201700090	MN122194	13.9	13-07-17	Doetinchem	Africa 3.3
AS201700096	MN122195	14.4	14-07-17	Rosmalen	Africa 3.3
AS201700103	MN122196	22.7	19-07-17	Gemonde	Europe 3
AS201700106	MN122197	23.8	25-07-17	Soest	Africa 3.3
AS201700109	MN122198	25.2	25-07-17	Bennekom	Europe 3
AS201700112	MN122199	18	27-07-17	Enschede	Africa 3.3
AS201700118	MN122200	22	03-08-17	Huizen	Africa 3.3
AS201700121	MN122201	27.8	01-08-17	Koekange	Africa 3.2
AS201700124	MN122202	16.4	01-08-17	Enschede	Africa 3.3
AS201700127	MN122203	26.6	30-07-17	Grenspad	Europe 3
AS201700130	MN122204	20.5	16-08-17	Lelystad	Africa 3.3
AS201700152	MN122205	24.98	19-07-17	IJsselstein	Africa 3.3
AS201700155	MN122206	15.13	10-08-17	Almere	Africa 3.2
AS201700167	MN122207	29.27	01-09-17	Almere	Africa 3.3
AS201700170	MN122208	26.03	25-08-17	Almere	Africa 3.3
AS201700174	MN122209	22.72	29-08-17	Utrecht	Africa 3.3
AS201700177	MN122210	21.85	15-09-17	Epe	Africa 3.3
AS201700186	MN122211	23.93	13-09-17	Zoeterwoude	Africa 3.2
AS201700189	MN122212	16.52	14-09-17	Eext	Africa 3.3
AS201700248	MN122213	23.65	20-09-17	Hardinxveld-Giessendam	Europe 3
AS201700254	MN122214	17.92	22-09-17	Hilversum	Africa 3.2
AS201800038	MN122215	17.75	11-09-17	Eastermar	Europe 3
AS201800081	MN122216	17.69	27-07-18	Naarden	Africa 3.3
AS201800082	MN122217	20.39	27-07-18	Naarden	Africa 3.3
AS201800084	MN122218	27.04	31-07-18	Spijk (West Betuwe)	Africa 3.3
AS201800086	MN122219	27.86	01-08-18	Middelburg	Africa 3.3
AS201800087	MN122220	21.72	01-08-18	Wierden	Africa 3.2
AS201800088	MN122221	18.60	01-08-18	Losser	Africa 3.3
AS201800089	MN122222	22.61	02-08-18	Rijswijk	Africa 3.3
AS201800090	MN122223	28.02	02-08-18	Ermelo	Africa 3.3
AS201800091	MN122224	17.73	02-08-18	Ermelo	Africa 3.2
AS201800092	MN122225	23.42	02-08-18	Rotterdam	Africa 3.3
AS201800093	MN122226	19.40	03-08-18	Leiden	Africa 3.3
AS201800094	MN122227	20.94	03-08-18	Bilthoven	Africa 3.2
AS201800095	MN122228	23.58	06-08-18	Middelburg	Africa 3.3
AS201800097	MN122229	28.93	03-08-18	Oldambt	Europe 3
AS201800099	MN122230	22.52	07-08-18	Noordosterpolder	Africa 3.2
AS201800100	MN122231	20.24	07-08-18	Venlo	Africa 3.2
AS201800101	MN122232	27.4	07-08-18	Tynaarlo	Africa 3.2
AS201800102	MN122233	20.81	09-08-18	Noordenveld	Africa 3.2
AS201800103	MN122234	22.19	10-08-18	Raalte	Africa 3.3
AS201800112	MN122235	16.36	14-08-18	Boekel	Europe 3
AS201800113	MN122236	15.97	14-08-18	Westland	Africa 3.2
AS201800114	MN122237	22.70	15-08-18	Noordoostpolder	Africa 3.3
AS201800115	MN122238	23.17	15-08-18	Midden Groningen	Africa 3.1
AS201800116	MN122239	17.15	16-08-18	Groningen	Africa 3.3
AS201800118	MN122240	25.68	16-08-18	Den Haag	Africa 3.2
AS201800120	MN122241	22.09	17-08-18	Utrechtse Heuvelrug	Africa 3.3
AS201800121	MN122242	17.80	17-08-18	Heerenveen	Africa 3.3
AS201800122	MN122243	21.00	21-08-18	Tytsjerksteradiel	Africa 3.3
AS201800123	MN122244	20.02	21-08-18	Tytsjerksteradiel	Africa 3.2
AS201800125	MN122245	25.03	22-08-18	Westerveld	Africa 3.2
AS201800126	MN122246	24.00	22-08-18	Hardenberg	Africa 3.2
AS201800127	MN122247	25.07	22-08-18	Groningen	Africa 3.3
AS201800128	MN122248	15.74	22-08-18	Zuidhorn	Africa 3.3
AS201800129	MN122249	21.44	23-08-18	Lochem	Africa 3.2
AS201800150	MN122250	23.67	30-08-18	Bloemendaal	Africa 3.3
AS201800151	MN122251	24.42	30-08-18	Bloemendaal	Africa 3.3
AS201800152	MN122252	20.87	30-08-18	Bloemendaal	Africa 3.3
AS201800154	MN122253	17.26	31-08-18	Oldenbroek	Africa 3.3
AS201800155	MN122254	16.16	31-08-18	Heerhugowaard	Africa 3.2
AS201800156	MN122255	17.99	31-08-18	Bosch en Duin	Africa 3.3
AS201800157	MN122256	22.89	04-09-18	Borger-Odoorn	Africa 3.3

### Phylogenetic analysis of USUV in the Netherlands

**Figure 1:**
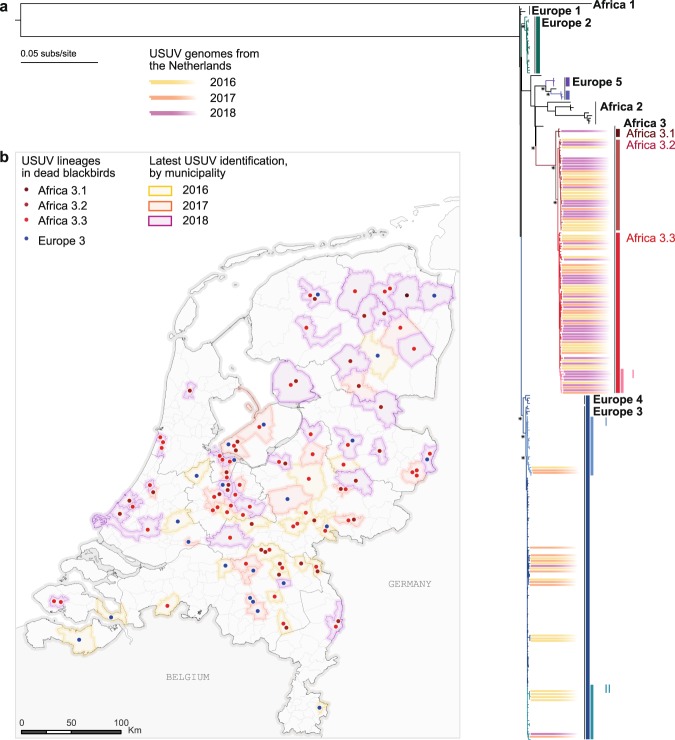
Phylogenetic analysis and geographic distribution of USUV strains detected in dead blackbirds in the Netherlands. (**a**) Maximum likelihood phylogeny of USUV complete coding sequences. PhyCLIP’s cluster designation is indicated in colour and asterisk indicate bootstrap scores ≥80%. (**b**) Geographic distribution of USUV clusters detected in dead blackbirds in the Netherlands. Dots indicate the center of municipality at which USUV positive dead birds were collected. In municipalities where more than one dead blackbird were collected, dots are dispersed and the municipality is colored according to year of most recent death. Scale bar represents units of substitutions per site.

Phylogenetic analysis revealed the co-circulation of USUV lineages Europe 3 and Africa 3 in the Netherlands (Fig. 1), with Africa 3 lineage viruses most frequently detected. The USUV lineage Africa 3 has been previously detected in Germany but not as major variant (9 of the 108 published whole genomes). USUV from blackbirds in the Netherlands are at this stage considered the most numerous within this lineage. In contrast, while the Europe 3 lineage was most commonly found to be associated with blackbird deaths in Germany between 2011 and 2016, this lineage appears to be less frequently detected in the Netherlands. Viruses belonging to the Europe 3 lineage formed several groups which were interspersed with viruses found in neighbouring countries. The two lineages were shown to co-circulate in the Netherlands during 3 consecutive years. In all three years, Africa 3 lineage viruses were more frequently detected (Table 2).

**Table 2:** Overview of the presence of the different USUV lineage during 2016–2018.

	USUV Africa 3 (%)	USUV Europe 3 (%)
2016	30 (69.8%)	13 (30.2%)
2017	19 (65.5%)	10 (34.5%)
2018	39 (95.1%)	2 (4.9%)

These differences in phylogenetic signal strongly suggests differences in the emergence of these two lineages in the Netherlands, with continuous circulation of the Africa 3 lineage and repeated introductions of viruses from the Europe 3 lineage. However, systematic sequencing of a representative set of samples from birds from other parts of Europe in the same time period is needed to resolve this question.

**Figure 2:**
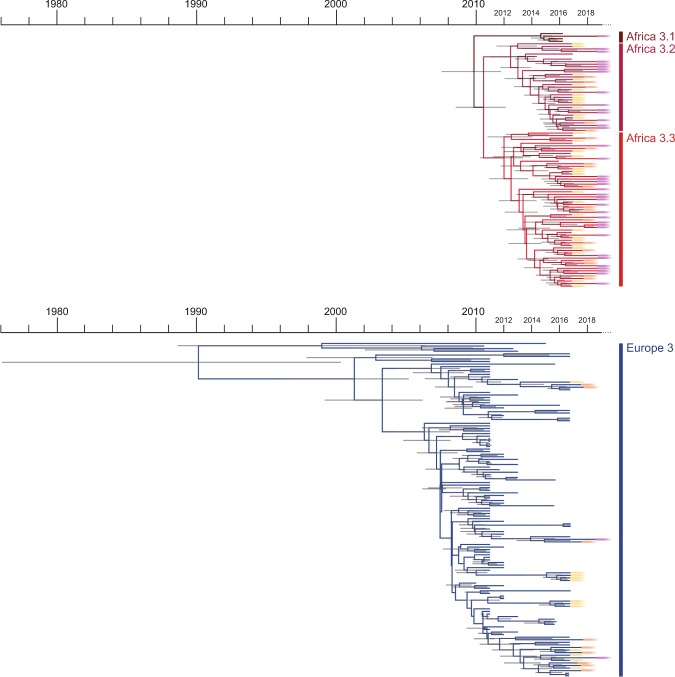
Molecular clock phylogeny of the complete coding sequences of USUV lineages detected in dead blackbirds from the Netherlands. Annotations correspond to clusters designed by PhyCLIP. Node bars indicate 95% confidence intervals of the time of the most common ancestor.

PhyCLIP^37^, a statistically-principled approach to delineate phylogenetic trees into clusters, was used to resolve the phylogenetic clustering of USUV (Fig. 1). PhyCLIP showed that the Africa 3 lineage can be divided into 3 different clusters: (1) a cluster comprising 3 sequences from Germany from 2016 and 1 sequence from the Netherlands from 2018 (2) a cluster comprising 32 USUV sequences detected in the Netherlands between 2016–2018, as well as 3 USUV sequences detected in Germany in 2014 and 2016, and (3) a cluster comprising 55 sequences detected in the Netherlands in 2016–2018 and 4 sequences detected in Germany and Belgium in 2015–2016, in this cluster 10 sequences could further be delineated into a nested sub-cluster. Delineation of these 3 clusters and the nested sub-cluster of the Africa 3 lineage does not appear to be structured over time (Fig. 2). Furthermore, sequences from the Netherlands from clusters Africa 3.2 and Africa 3.3 have been detected from across the country each year, while cluster USUV Africa 3.1 was only detected once in 2018. PhyCLIP delineated the Europe 3 USUV lineage as one clade with two different sub-clusters: (I) a sub-cluster with sequences detected in Belgium, Germany and the Netherlands in 2011–2017 and (II) a sub-cluster detected in Belgium, Germany and the Netherlands in 2011–2018. Also for the Europe 3 lineage, delineation of the sub-clusters does not appear to be structured over time. Four sequences from blackbirds and mosquitos detected in Italy that were previously classified as a distinct clade, Europe 4^24^, were classified by PhyCLIP within the Europe 3 lineage.

Apart from the Europe 3 lineage and the 3 clusters within the Africa 3 lineage, PhyCLIP identified 3 additional clusters. The Europe 2 lineage, which consists of sequences identified in humans, blackbirds and mosquitos in Italy, Austria and Hungary between 2001 and 2017, was recognized as a cluster. PhyCLIP delineated two higher resolution clusters from the Europe 5 lineage, the first consisting of sequences from mosquitos in Israel from 2004, the second consisting of sequences from mosquitos in Israel from 2015. Several USUV sequences could not be classified into clusters. This suggests that more lineages might be circulating, but that the number of sequences available at the moment is too limited to enable delineation into clusters by PhyCLIP.

An overview of the geographical distribution of the USUV clusters detected in dead blackbirds in the Netherlands is displayed in Fig. 1 and in the Supplementory Movie. The first set of dead blackbirds positive for USUV collected in September 2016, were found in 10 of 12 different provinces, indicating already widespread circulation at the time of detection. The USUV identified from this first set were genetically diverse and each of the four USUV clusters identified in the Nertherlands were already present at that time. The different USUV clusters all have a widespread geographic distribution throughout the Netherlands.

### Molecular clock phylogeny of USUV lineages detected in the netherlands

BEAST analysis was performed to determine the time to most recent common ancestor of the USUV Africa 3 and Europe 3 lineages, both detected in the Netherlands. Separate BEAST analyses were performed for each of the two lineages. The estimated time to the most recent common ancestor of lineage Africa 3 was shown to be around 2009 (between 2007–2012, 95% confidence interval). The estimated time to the most recent common ancestor of the Europe 3 lineage, encompassing USUV sequences from the Netherlands, was shown to be around 2002 (1996–2006, 95% confidence interval).

## Discussion

The emergence of USUV in Europe is causing massive die-offs of mainly common blackbirds. Besides being of concern for wildlife conservation, the occurrence of numerous and sustained outbreaks in wildlife should be considered as a serious warning signal from the perspective of surveillance for zoonotic pathogens. The increasing numbers of reports of asymptomatic human USUV infections as well as cases of mild to severe neuroinvasive USUV infections in humans may be due to changes in awareness and surveillance but may also be an effect of increased human exposure to this zoonotic risk. Dense genomic monitoring of viral pathogens supports outbreak investigations by providing insights to paterns of transmission. Since USUV was only recently recognized in the Netherlands, we used genomic sequencing to gain insight in how this emerging arbovirus spread and evolve in a previously naïve population. We describe the genetic characterization of USUV genomes from tissue samples from dead blackbirds in the Netherlands throughout 3 consecutive years. An amplicon-based sequencing approach using Nanopore sequencing was used to monitor the genetic diversity of USUV^36^. This protocol proved to be sensitive enough to sequence the vast majority of tissue samples from dead blackbird surveillance. This study shows that genomic sequencing on the Nanopore platform is a powerfull approach for monitoring and tracing ongoing arbovirus infections in dead wildlife.

The USUV Africa 3 lineage was found to be predominantly circulating in the Netherlands. Furthermore, divergence inside this lineage was shown to justify its division into 3 higher resolution clusters. The absence of clear temporal delineation in the phylogeny of the USUV Africa 3 lineage could indicate that subsequent to the first identification of USUV in the Netherlands in 2016, the outbreaks in 2017 and 2018 were more likely caused by USUV lineages that persisted during winter in the Netherlands, rather than by repeated introduction of USUV strains from areas outside the Netherlands. It remains unclear if and how USUV can persist in the Netherlands during winter time, whether through vertical tranmission in mosquitoes, maintainance in birds or overwintering mosquitoes, or if it remains present in other animal species. Therefore, extending the diversity of species included in USUV surveillance efforts would be a useful consideration. A recent study in Germany also showed an increase in the detection of the USUV Africa 3 lineage in 2017 and 2018^26^, however only partial sequence data is available and more genetic information is needed before it can accurately be used in phylogenetic analysis. The results differed for the USUV Europe 3 lineage: this lineage was less frequently detected in the Netherlands, and sequences from the Netherlands were interspersed with viruses from Germany and Belgium. The reason for this is unknown but might suggest that the USUV Europe 3 lineage – unlike the USUV Africa 3 lineage – is not enzootic in the Netherlands but periodically introduced from neighbouring countries.

USUV was detected in the Netherlands through bird mortality in 2016, but the most recent common ancestors of both the Africa 3 lineage and Europe 3 lineage are estimated to be well before the initial detection. The two time windows identified are broad, and more data from other regions and previous years has to be produced to generate more precise estimates. However, this information, taken together with the described diversity of USUV detected in the Netherlands since the beginning of the outbreak in September 2016, suggests limited circulation of different USUV lineages in the Netherlands before its initial detection. USUV circulation may have been boosted in 2016 by favourable environmental conditions for mosquitoes^28,30^. Alternatively, several introductions from neighbouring countries could have occurred close to the date of first detection and spread efficiently with these favourable conditions. Phylogenetic and molecular clock analyses conducted for the reconstrution of the Zika virus epidemic in the Americas have estimated the time of introduction of Zika virus more than a year before it was detected through human-disease surveillance^35^. It is unclear whether blackbird mortality associated with USUV infection did not occur prior to 2016 or whether it occurred at levels that did not prompt detection through citizen science-based alerting system. In October 2012, 66 songbirds, including 34 blackbirds, were found dead throughout the Netherlands but all tested negative for USUV^38^. Serological testing of a small number of serum samples from birds in the Netherlands in 2015 did show a seroprevalence of 2.8% for USUV; however, the positive birds consisted of species that are partially migratory and therefore this result does not necessarly indicate local circulation of the virus at that time^39^. Retrospective testing of other samples for the presence of USUV may help to elucidate this. In addition, care has to be taken with the interpretation of the BEAST analysis since recently it was shown that by sequencing an ancient hepatitis B virus the evolutionary rate of the virus has shown to be different than previously thought^40^. To date, only very recent data on USUV is available and we do not know how the virus behaves over a prolonged period of time.

Several USUV sequences remained unclassified by the statistically-principled approach for phylogenetic clustering (PhyCLIP^37^). This indicates that more lineages might be present, but that the amount of genetic data available is at the moment too limited to enable accurate phylogenetic clustering. In addition, by adding a large amount of sequences to the public database, the genomic information currently available is very skewed towards the USUV strains circulating in the Netherlands, Germany and Italy. Recent identification and generation of USUV genomic data outside Europe (Senegal^5^ and Israel^41^) have had an important impact on the understanding of the global USUV diversity. To get a better overview of USUV diversity in Europe and a better understanding of USUV evolution, it is important that more sequence data is generated from other countries. Given the detection of USUV in the Netherlands, it is important to identify the vector competence in local mosquitos, although a previous studie has already shown the vector competence in the *Culex pipiens* mosquito which is present in the Netherlands^3^.

This study describes a proactive investigation of USUV emergence and spread in the Netherlands through a national wildlife disease scanning surveillance programme associated with the application of a protocol enabling fast and accurate complete genome sequencing of USUV on the Nanopore platform. We add knowledge relating to the USUV epidemiology and describe the genomic diversity profile of USUV circulating in the country. Our analysis suggests that USUV is likely circulating between neighboring countries in Western Europe, where it has been established and is overwintering. We report an important genomic diversity of the viruses circulating in the Netherlands, observable already at the time of the first identifications of USUV in the country. We highlight issues of bias in surveillance, as well as the possibility of eventual silent circulation of the virus in European countries preceding epizootics detection. Another virus circulating in Europe, West-Nile virus, has antigenic cross-reactivity, similar transmission cycle and may interact at population level with USUV^42^. With the first report of West Nile virus in Germany in 2018^43^, the detection of authochthonous West Nile virus in the Netherlands is likely a matter of time. The readily established dead wildlife disease surveillance programme presented here and the expansion of the described protocol for sequencing on the Nanopore platform to West Nile virus should in this scenario allow for the real time monitoring of eventual reciprocal interaction in the dynamics of West Nile virus.

## Methods

### Dead blackbirds surveillance

Mortality in blackbirds was reported through a citizen science-based alerting system. For a selection of the cases reported, dead blackbirds were collected, autopsy was performed, and brain tissues were sampled for USUV diagnostics if autopsy indicated USUV as the possible cause of death until the first detection of USUV in 2016^30^, and systematically afterwards. The selection was based on freshness of the carcasses and on geographic location, with oversampling at locations where USUV activity had not been identified by then. Therefore, the sampling reflects the edges of observed bird mortality rather than the local evolution of the virus over time. Locations where dead blackbirds were found were registered by municipality, and date of death was based on the date of the sample collection.

### Usutu virus diagnostics

Tissue from dead blackbirds was homogenized using the Fastprep bead beater (4.0 m/s for 20 seconds). Samples were spun down for 10 minutes at 10.000 x*g* after which Phocine distemper virus (PDV) was added as internal NA extraction control to the supernatant and total NA was extracted from the supernatant using the Roche MagNA Pure. The NA was screened for the presence of USUV using real-time PCRs described by Nicolay *et al*.^44^ and Jost *et al*.^45^, in duplex with PDV.

### Multiplex PCR for nanopore sequencing

The multiplex PCR for MinION sequencing was performed as previously described^36^. In short, random primers (Invitrogen) were used to perform reverse transcription using ProtoScript II (NEB, cat. no. E6569) after which USUV specific multiplex PCR was performed in 2 reactions using Q5 Hot Start High-Fidelity DNA Polymerase (NEB, cat no. M0493). Nanopore sequencing was performed according to manufacturer’s instructions using the 1D Native barcoding genomic DNA Kit (Nanopore, EXP-NBD103 and SQK-LSK108) on a FLO-MIN106 flowcell. A total of 12 or 24 samples were multiplexed per sequence run.

### Data analysis MinION sequencing

Raw sequence data was demultiplexed using Porechop (https://github.com/rrwick/porechop). Primers were trimmed and reads were quality controlled to a minimal length of 150 and a median PHRED score of 10 using QUASR^46^. A reference based alignment against an arbitrary chosen Usutu virus genome was performed in Geneious^47^ or in CLC Genomic Workbench 11.0 (https://www.qiagenbioinformatics.com/). The consensus genome was extracted and compared to the non-redundant database using Blastn^48^. The most closely related sequence was selected and used for a second reference based alignment using the quality controlled reads. The consensus genome was extracted and positions with a coverage <100 were replaced with an “N”. This threshold was set based on a recent publication demonstrating that by using this threshold the quality of full genomes is very high (one position per 106 USUV whole genomes sequences sequenced is called erroneously)^36^. Homopolymeric regions were manually checked and resolved consulting the closest reference genome.

### Phylogenetic analysis

All available full length USUV genomes were retrieved from GenBank^49^ on 12 February 2019 and aligned with the newly obtained USUV sequences using MUSCLE^50^. Sequences with >20% “Ns” were not included in the phylogenetic analysis. The alignment was manually checked for discrepancies after which IQ-TREE^51^ was used to perform maximum likelihood phylogenetic analysis under the GTR + I + G4 model as best predicted model using the ultrafast bootstrap option with 1,000 replicates.

### PhyCLIP

PhyCLIP^37^ was used to delineate the phylogenetic tree in clusters. The default settings for intermediate-resolution clustering were used with the recommended range of input parameters to determine the optimal parameters: S = 3–10 (increasing by 1), FDR = 0.05–0.20 (increasing by 0.05) and gamma = 1–3 (increasing by 0.5).

### BEAST analysis

Bayesian phylogenetic trees were inferred using BEAST v1.10.3^52^. The HKY site model was used with 4 gamma categories with 3 partition into codon positions to generate an uncorrelated relaxed molecular clock. The tree prior was set to exponential growth and random sampling for the Africa 3 lineage and to constant size for the Europe 3 lineage. MCMC was set to 80,000,000 generations for both lineages. Log files were analyzed in Tracer v1.7.1 to check if ESS values were beyond threshold (>200). Tree annotator v1.8.4 was used with 10% burnin and median node heights. The tree was annotated and visualized using FigTree^53^.

### Geographical distribution of USUV strains detected in dead blackbirds in the Netherlands

The map was created using ArcGIS 10.6 software (ESRI Inc., Redlands, CA, USA) (http://www.esri.com/) and uses the datasets *Gemeentegrenzen 2019* and *Provinciegrenzen 2019* by ESRI Nederland (services.arcgis.com) for administrative boundaries.

## Supplementary information

Supplementary information.

Supplementary Movie: Geographic distribution of USUV strains detected in dead blackbirds in the Netherlands.

## Data Availability

The genomic sequences of the Usutu viruses sequenced in this study have been deposited in the GenBank database under the accession numbers MN122145 – M122256.
