# Supplementary material for: Genomic monitoring to understand the emergence and spread of Usutu virus in the Netherlands, 2016–2018
Source: Sci Rep. 2020 Feb 18;10:2798. doi: 10.1038/s41598-020-59692-y (PMC7029044; doi:10.1038/s41598-020-59692-y)
Supplement: Supplementary file 1 — Supplementary information. [file 41598_2020_59692_MOESM1_ESM.pdf]

Genomic monitoring to understand the emergence and spread of Usutu virus in the Netherlands, 2016-2018

B.B. Oude Munnink[1*^], E. Münger[1*], D.F. Nieuwenhuijse[1], R. Kohl[1], A. van der Linden[1], C.M.E Schapendonk[1], H. van der Jeugd[2], M. Kik[3,4], J. M. Rijks[3], C.B.E.M. Reusken[1,5*] and M. Koopmans[1*]

Affiliations

[1] ErasmusMC, Department of Viroscience, WHO collaborating centre for arbovirus and viral hemorrhagic fever Reference and Research, Rotterdam, the Netherlands

[2] Vogeltrekstation - Dutch Centre for Avian Migration and Demography, NIOO-KNAW, Wageningen, the Netherlands

[3] Dutch Wildlife Health Centre, University of Utrecht, the Netherlands

[4] Department of Pathobiology, Pathology, Faculty of Veterinary Medicine, Utrecht University, the Netherlands

[5] Current co-affiliate: Centre for Infectious Disease Control, National Institute for Public Health and the Environment, Bilthoven, the Netherlands

[*] These authors contributed equally to this manuscript

[^] Corresponding author: (b.oudemunnink@erasmusmc.nl)

Supplementory Files

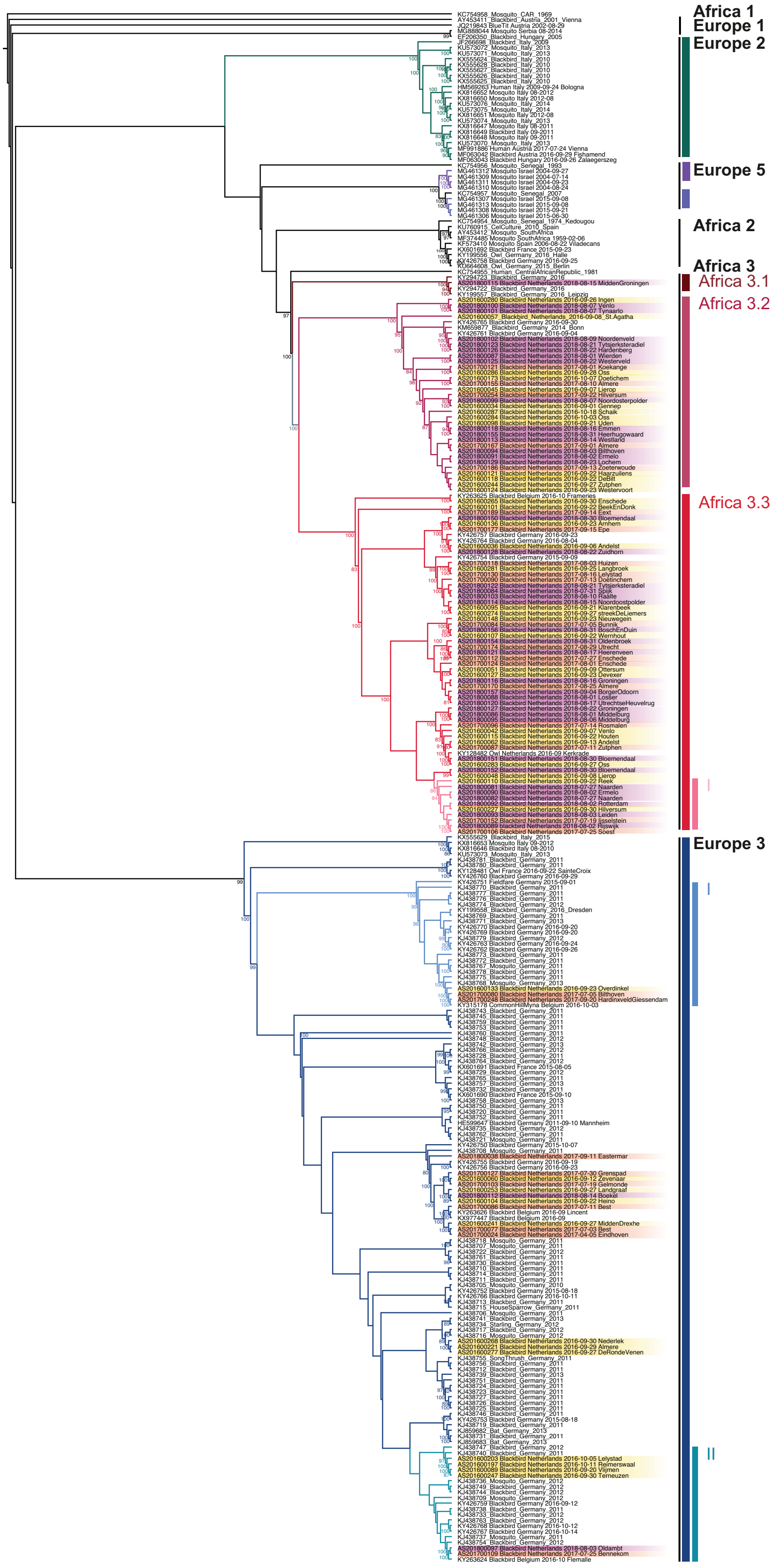

Supplementary figure 1: **Maximum likelihood phylogeny of USUV complete coding region sequences.** Bootstrap scores (≥80%) are shown next to well-supported node. Tip labels are highlighted according to year of death (yellow: 2016, orange: 2017, red: 2018). Branches are transformed proportionally for clarity reasons.

Supplementary movie 1: Geographic distribution of USUV strains detected in dead blackbirds in the Netherlands. Dots are colored according to the USUV cluster detected.

Supplementary table 1: Detailed overview of the different USUV blackbird samples sequenced during this study. Overview of the number of reads generated, the percentage of USUV reads and the proportion of successfully sequenced amplicons

Sample ID	Accession number	Ct Value	Collection date	Location	USUV lineage	No. of reads	Number USUV reads	% USUV reads	% Genome coverage
AS201600034	MN122145	19,45	01-09-2016	Gennep	Africa 3.2	40.010	37.841	94,58%	100,00%
AS201600036	MN122146	15,38	06-09-2016	Andelst	Africa 3.3	254.841	235.629	92,46%	100,00%
AS201600042	MN122147	14,21	07-09-2016	Venlo	Africa 3.3	396.530	364.347	91,88%	94,51%
AS201600045	MN122148	14,97	07-09-2016	Lierop	Africa 3.2	273.363	243.520	89,08%	100,00%
AS201600048	MN122149	19,74	07-09-2016	Lierop	Africa 3.3	450.846	433.660	96,19%	100,00%
AS201600051	MN122150	21,44	07-09-2016	Ottersum	Africa 3.3	59.699	55.614	93,16%	100,00%
AS201600057	MN122151	15,6	08-09-2016	Sint Agatha	Africa 3.2	206.154	196.488	95,31%	100,00%
AS201600060	MN122152	19,75	12-09-2016	Zevenaar	Europe 3	181.639	171.876	94,63%	100,00%
AS201600062	MN122153	16,99	13-09-2016	Andelst	Africa 3.3	351.520	324.641	92,35%	100,00%
AS201600089	MN122154	21,08	20-09-2016	Vlijmen	Europe 3	436.160	425.473	97,55%	100,00%
AS201600095	MN122155	19,78	21-09-2016	Klarenbeek	Africa 3.3	113.366	108.617	95,81%	100,00%
AS201600098	MN122156	20,73	21-09-2016	Uden	Africa 3.2	459.499	422.739	92,00%	100,00%
AS201600101	MN122157	20,34	22-09-2016	Beek en Donk	Africa 3.3	214.104	206.415	96,41%	100,00%
AS201600104	MN122158	18,92	22-09-2016	Heino	Europe 3	243.265	232.577	95,61%	100,00%
AS201600107	MN122159	21,85	22-09-2016	Wernhout	Africa 3.3	67.923	65.310	96,15%	100,00%
AS201600110	MN122160	24,21	22-09-2016	Reek	Africa 3.3	385.874	361.013	93,56%	100,00%
AS201600115	MN122161	24,06	22-09-2016	Houten	Africa 3.3	233.497	216.505	92,72%	100,00%
AS201600118	MN122162	21,02	22-09-2016	De Bilt	Africa 3.2	358.807	334.651	93,27%	100,00%
AS201600121	MN122163	20,39	22-09-2016	Haarzuilens	Africa 3.2	287.872	258.695	89,86%	100,00%
AS201600124	MN122164	23,8	23-09-2016	Westervoort	Africa 3.2	653.893	599.948	91,75%	100,00%
AS201600127	MN122165	17,47	23-09-2016	Deventer	Africa 3.3	427.978	392.524	91,72%	100,00%
AS201600133	MN122166	22,59	23-09-2016	Overdinkel	Europe 3	313.623	288.572	92,01%	100,00%
AS201600136	MN122167	16,99	23-09-2016	Arnhem	Africa 3.3	148.532	138.454	93,21%	100,00%
AS201600148	MN122168	24,79	23-09-2016	Nieuwegein	Africa 3.3	205.086	180.965	88,24%	100,00%
AS201600173	MN122169	20,33	07-10-2016	Doetichem	Africa 3.2	366.043	336.169	91,84%	100,00%

AS201600197	MN122170	20,13	11-10-2016	Reimerswaal	Europe 3	445.535	415.996	93,37%	100,00%
AS201600203	MN122171	32,01	05-10-2016	Lelystad	Europe 3	395.173	179.137	45,33%	86,64%
AS201600221	MN122172	19,95	22-09-2016	Haarzuilens	Europe 3	254.400	243.169	95,59%	100,00%
AS201600227	MN122173	26,23	30-09-2016	Hilversum	Africa 3.3	438.874	374.117	85,24%	100,00%
AS201600241	MN122174	20,16	27-09-2016	Midden-Drenthe	Europe 3	389.453	332.443	85,36%	100,00%
AS201600244	MN122175	18,89	27-09-2016	Zutphen	Africa 3.2	470.128	445.731	94,81%	100,00%
AS201600247	MN122176	14,07	30-09-2016	Terneuzen	Europe 3	463.113	440.036	95,02%	100,00%
AS201600253	MN122177	21,51	27-09-2016	Landgraaf	Europe 3	437.136	413.027	94,48%	100,00%
AS201600265	MN122178	16,48	30-09-2016	Enschede	Africa 3.3	420.124	399.179	95,01%	100,00%
AS201600268	MN122179	16,72	30-09-2016	Nederlek	Europe 3	406.571	378.076	92,99%	100,00%
AS201600274	MN122180	21,11	27-09-2016	streek De Liemers	Africa 3.3	466.939	427.548	91,56%	100,00%
AS201600277	MN122181	17,43	27-09-2016	De Ronde Venen	Europe 3	267.079	248.174	92,92%	100,00%
AS201600280	MN122182	21,87	26-09-2016	Ingen	Africa 3.2	363.218	337.978	93,05%	100,00%
AS201600281	MN122183	14,07	25-09-2016	Langbroek	Africa 3.3	532.467	505.856	95,00%	100,00%
AS201600283	MN122184	19,94	27-09-2016	Oss	Africa 3.3	501.407	469.245	93,59%	100,00%
AS201600284	MN122185	20,09	03-10-2016	Oss	Africa 3.2	492.022	462.800	94,06%	100,00%
AS201600286	MN122186	28,48	28-09-2016	Oss	Africa 3.2	381.449	277.571	72,77%	100,00%
AS201600287	MN122187	14,46	27-09-2016	Schaik	Africa 3.2	302.225	284.593	94,17%	100,00%
AS201700024	MN122188	19,88	11-04-2017	Westvoort	Europe 3	2.202.744	2.052.733	93,19%	100,00%
AS201700077	MN122189	16,8	03-07-2017	Best	Europe 3	79.111	76.327	96,48%	100,00%
AS201700080	MN122190	19,6	05-07-2017	Bilthoven	Europe 3	213.003	206.206	96,81%	100,00%
AS201700084	MN122191	16,6	05-07-2017	Bunnik	Africa 3.3	418.412	403.924	96,54%	100,00%
AS201700086	MN122192	18,3	11-07-2017	Best	Europe 3	145.556	403.924	277,50%	100,00%
AS201700087	MN122193	17	11-07-2017	Zutphen	Africa 3.3	380.108	356.528	93,80%	100,00%
AS201700090	MN122194	13,9	13-07-2017	Doetinchem	Africa 3.3	212.170	193.097	91,01%	100,00%
AS201700096	MN122195	14,4	14-07-2017	Rosmalen	Africa 3.3	256.479	232.270	90,56%	100,00%
AS201700103	MN122196	22,7	19-07-2017	Gemonde	Europe 3	479.852	442.595	92,24%	100,00%
AS201700106	MN122197	23,8	25-07-2017	Soest	Africa 3.3	379.681	352.017	92,71%	100,00%
AS201700109	MN122198	25,2	25-07-2017	Bennekom	Europe 3	304.296	279.945	92,00%	100,00%
AS201700112	MN122199	18	27-07-2017	Enschede	Africa 3.3	194.224	175.633	90,43%	100,00%
AS201700118	MN122200	22	03-08-2017	Huizen	Africa 3.3	305.646	280.816	91,88%	100,00%
AS201700121	MN122201	27,8	01-08-2017	Koekange	Africa 3.2	347.015	327.754	94,45%	100,00%
AS201700124	MN122202	16,4	01-08-2017	Enschede	Africa 3.3	213.519	195.685	91,65%	100,00%
AS201700127	MN122203	26,6	30-07-2017	Grenspad	Europe 3	458.254	430.715	93,99%	100,00%
AS201700130	MN122204	20,5	16-08-2017	Lelystad	Africa 3.3	243.535	219.390	90,09%	100,00%
AS201700152	MN122205	24,98	19-07-2017	IJsselstein	Africa 3.3	705.980	666.427	94,40%	100,00%
AS201700155	MN122206	15,13	10-08-2017	Almere	Africa 3.2	867.951	830.630	95,70%	100,00%
AS201700167	MN122207	29,27	01-09-2017	Almere	Africa 3.3	110.557	91.170	82,46%	97,89%
AS201700170	MN122208	26,03	25-08-2017	Almere	Africa 3.3	771.864	720.961	93,41%	100,00%
AS201700174	MN122209	22,72	29-08-2017	Utrecht	Africa 3.3	466.475	443.038	94,98%	100,00%
AS201700177	MN122210	21,85	15-09-2017	Epe	Africa 3.3	433.446	418.002	96,44%	100,00%
AS201700186	MN122211	23,93	13-09-2017	Zoeterwoude	Africa 3.2	391.377	378.852	96,80%	100,00%
AS201700189	MN122212	16,52	14-09-2017	Eext	Africa 3.3	601.308	590.793	98,25%	100,00%

AS201700248	MN122213	23,65	20-09-2017	Hardinxveld-Giessendam	Europe 3	882.815	866.286	98,13%	100,00%
AS201700254	MN122214	17,92	22-09-2017	Hilversum	Africa 3.2	620.452	612.027	98,64%	100,00%
AS201800038	MN122215	17,75	11-09-2017	Eastermar	Europe 3	299.889	296.586	98,90%	100,00%
AS201800081	MN122216	17,69	27-07-2018	Naarden	Africa 3.3	201.765	186.011	92,19%	100,00%
AS201800082	MN122217	20,39	27-07-2018	Naarden	Africa 3.3	164.990	151.905	92,07%	100,00%
AS201800084	MN122218	27,04	31-07-2018	Spijk (West Betuwe)	Africa 3.3	187.723	170.358	90,75%	100,00%
AS201800086	MN122219	27,86	01-08-2018	Middelburg	Africa 3.3	130.755	118.471	90,61%	100,00%
AS201800087	MN122220	21,72	01-08-2018	Wierden	Africa 3.2	263.219	239.759	91,09%	100,00%
AS201800088	MN122221	18,60	01-08-2018	Losser	Africa 3.3	124.856	115.675	92,65%	100,00%
AS201800089	MN122222	22,61	02-08-2018	Rijswijk	Africa 3.3	186.351	175.797	94,34%	100,00%
AS201800090	MN122223	28,02	02-08-2018	Ermelo	Africa 3.3	206.888	188.010	90,88%	100,00%
AS201800091	MN122224	17,73	02-08-2018	Ermelo	Africa 3.2	191.812	180.871	94,30%	100,00%
AS201800092	MN122225	23,42	02-08-2018	Rotterdam	Africa 3.3	123.066	117.020	95,09%	100,00%
AS201800093	MN122226	19,40	03-08-2018	Leiden	Africa 3.3	140.942	134.495	95,43%	100,00%
AS201800094	MN122227	20,94	03-08-2018	Bilthoven	Africa 3.2	597.892	580.684	97,12%	100,00%
AS201800095	MN122228	23,58	06-08-2018	Middelburg	Africa 3.3	210.387	203.003	96,49%	100,00%
AS201800097	MN122229	28,93	03-08-2018	Oldambt	Europe 3	266.655	229.877	86,21%	97,34%
AS201800099	MN122230	22,52	07-08-2018	Noordosterpolder	Africa 3.2	144.958	141.853	97,86%	100,00%
AS201800100	MN122231	20,24	07-08-2018	Venlo	Africa 3.2	253.288	249.075	98,34%	100,00%
AS201800101	MN122232	27,4	07-08-2018	Tynaarlo	Africa 3.2	442.866	425.879	96,16%	100,00%
AS201800102	MN122233	20,81	09-08-2018	Noordenveld	Africa 3.2	528.607	519.205	98,22%	100,00%
AS201800103	MN122234	22,19	10-08-2018	Raalte	Africa 3.3	256.294	252.060	98,35%	100,00%
AS201800112	MN122235	16,36	14-08-2018	Boekel	Europe 3	435.437	427.323	98,14%	100,00%
AS201800113	MN122236	15,97	14-08-2018	Westland	Africa 3.2	416.013	409.277	98,38%	100,00%
AS201800114	MN122237	22,70	15-08-2018	Noordoostpolder	Africa 3.3	328.036	317.647	96,83%	100,00%
AS201800115	MN122238	23,17	15-08-2018	Midden Groningen	Africa 3.1	261.869	247.512	94,52%	100,00%
AS201800116	MN122239	17,15	16-08-2018	Groningen	Africa 3.3	459.144	450.702	98,16%	100,00%
AS201800118	MN122240	25,68	16-08-2018	Den Haag	Africa 3.2	681.112	645.628	94,79%	100,00%
AS201800120	MN122241	22,09	17-08-2018	Utrechtse Heuvelrug	Africa 3.3	659.508	639.906	97,03%	100,00%
AS201800121	MN122242	17,80	17-08-2018	Heerenveen	Africa 3.3	516.450	504.976	97,78%	100,00%
AS201800122	MN122243	21,00	21-08-2018	Tytsjerksteradiel	Africa 3.3	416.489	404.015	97,00%	100,00%
AS201800123	MN122244	20,02	21-08-2018	Tytsjerksteradiel	Africa 3.2	270.594	263.338	97,32%	100,00%
AS201800125	MN122245	25,03	22-08-2018	Westerveld	Africa 3.2	527.696	509.063	96,47%	100,00%
AS201800126	MN122246	24,00	22-08-2018	Hardenberg	Africa 3.2	463.225	449.932	97,13%	100,00%
AS201800127	MN122247	25,07	22-08-2018	Groningen	Africa 3.3	460.008	438.914	95,41%	100,00%
AS201800128	MN122248	15,74	22-08-2018	Zuidhorn	Africa 3.3	374.471	371.035	99,08%	100,00%
AS201800129	MN122249	21,44	23-08-2018	Lochem	Africa 3.2	363.990	356.906	98,05%	100,00%
AS201800150	MN122250	23,67	30-08-2018	Bloemendaal	Africa 3.3	286.767	276.889	96,56%	100,00%
AS201800151	MN122251	24,42	30-08-2018	Bloemendaal	Africa 3.3	549.326	530.730	96,61%	100,00%
AS201800152	MN122252	20,87	30-08-2018	Bloemendaal	Africa 3.3	537.086	521.579	97,11%	100,00%
AS201800154	MN122253	17,26	31-08-2018	Oldenbroek	Africa 3.3	519.146	506.212	97,51%	100,00%
AS201800155	MN122254	16,16	31-08-2018	Heerhugowaard	Africa 3.2	242.133	236.605	97,72%	100,00%

AS201800156	MN122255	17,99	31-08-2018	Bosch en Duin	Africa 3.3	373.410	361.638	96,85%	100,00%
AS201800157	MN122256	22,89	04-09-2018	Borger-Odoorn	Africa 3.3	437.003	422.010	96,57%	100,00%
